# Experimental depletion of CD8^+ ^cells in acutely SIVagm-Infected African Green Monkeys results in increased viral replication

**DOI:** 10.1186/1742-4690-7-42

**Published:** 2010-05-11

**Authors:** Thaidra Gaufin, Ruy M Ribeiro, Rajeev Gautam, Jason Dufour, Daniel Mandell, Cristian Apetrei, Ivona Pandrea

**Affiliations:** 1 Division of Microbiology, Tulane National Primate Research Center, Covington LA, 70433, USA; 2 Theoretical Biology and Biophysics Group, Los Alamos National Laboratory, Los Alamos, NM 87544, USA; 3 Division of Veterinary Sciences, Tulane National Primate Research Center, Covington LA, 70433, USA; 4 Division of Microbiology, Tulane National Primate Research Center, Covington LA, 70433, USA and Center for Vaccine Research, University of Pittsburgh, Pittsburgh, PA 15261, USA; 5 Division of Comparative Pathology, Tulane National Primate Research Center, Covington LA, 70433 and Center for Vaccine Research, University of Pittsburgh, Pittsburgh, PA 15261, USA

## Abstract

**Background:**

*In vivo *CD8^+ ^cell depletions in pathogenic SIV infections identified a key role for cellular immunity in controlling viral load (VL) and disease progression. However, similar studies gave discordant results in chronically-infected SMs, leading some authors to propose that in natural hosts, SIV replication is independent of cellular immunity. To assess the role of cellular immune responses in the control of SIV replication in natural hosts, we investigated the impact of CD8^+ ^cell depletion during acute SIV infection in AGMs.

**Results:**

Nine AGMs were infected with SIVagm.sab and were followed up to day 225 p.i. Four were intravenously infused with the cM-T807 antibody on days 0 (50 mg/kg), 6, and 13 (10 mg/kg, respectively) post infection (p.i.). CD8^+ ^cells were depleted for up to 28 days p.i. in peripheral blood and LNs in all treated AGMs. Partial CD8^+ ^T cell depletion occurred in the intestine. SIVagm VLs peaked at similar levels in both groups (10^7^-10^8 ^RNA copies/ml). However, while VLs were controlled in undepleted AGMs, reaching set-point levels (10^4^-10^5 ^RNA copies/ml) by day 28 p.i., high VLs (>10^6 ^RNA copies/ml) were maintained by day 21 p.i. in CD8-depleted AGMs. By day 42 p.i., VLs were comparable between the two groups. The levels of immune activation and proliferation remained elevated up to day 72 p.i. in CD8-depleted AGMs and returned to preinfection levels in controls by day 28 p.i. None of the CD8-depleted animals progressed to AIDS.

**Conclusion:**

CD8^+ ^cells are responsible for a partial control of postacute viral replication in SIVagm.sab-infected AGMs. In contrast to macaques, the SIVagm-infected AGMs are able to control viral replication after recovery of the CD8^+ ^T cells and avoid disease progression.

## Background

African non-human primates (NHPs) have been infected with SIV for tens of thousands of years and this long term infection has resulted in the co-existence of virus and host [1,2]. The mechanisms by which African NHPs, such as AGMs, SMs and mandrills, prevent SIV disease progression to AIDS are not completely understood. During the recent years, data have rapidly accumulated in this field allowing the characterization of the pathogenesis of SIV infection in natural hosts. These studies have established three quintessential characteristics of SIV infection in natural hosts. First, the lack of disease progression is not due to an exquisite control of viral replication, as SIV VLs in chronically-infected African NHP hosts are in the same range or higher than in HIV-infected patients [3,4]. However, in contrast to pathogenic SIV and HIV infections, during chronic SIV infection in natural hosts, VLs are remarkably stable for long periods of time, suggesting an immune control of viral replication. Second, the lack of disease progression is not due to a lack of pathogenicity of SIVs in their natural host, as there is a significant depletion of peripheral and mucosal CD4^+ ^T cells during the acute phase of infection [5]. However, in stark contrast to pathogenic SIV and HIV infections, CD4^+ ^T cells are then restored during the chronic SIV infection in natural hosts [5]. Peripheral CD4^+ ^T cells rebound to near pre-infection levels [2-10]. In the intestine, however, CD4^+ ^T cells are only partially, albeit significantly, restored [5]. Finally, natural hosts of SIVs have significantly lower levels of CD4^+ ^CCR5^+ ^cells in blood, LNs, and mucosal tissues [1]. This may significantly impact the homing of activated, memory CD4^+ ^T cells to the intestine and, as a consequence, the efficacy of mucosal transmission of SIVs in these species [1,11]. Altogether, these characteristics define the paradox of SIV infection in natural hosts in which, in spite of low levels of cells susceptible to SIV infection (CCR5^+ ^CD4^+ ^T cells), there is a robust viral replication which does not substantially affect the homeostasis of CD4^+ ^T cells. During the recent years, based on these results, a new paradigm of SIV infection occurred, in which the preservation of CD4^+ ^T cells in natural hosts is mainly due to their ability to maintain normal levels of T cell immune activation, proliferation, and apoptosis [2,5,10,12,13]. This paradigm is supported by our recent observation that induction of immune activation in natural hosts of SIVs results in significant increases of CCR5 expression by CD4+ T cells, which fuel viral replication and result in CD4^+ ^T cell depletion [14]. Therefore, the current view is that the control of immune activation and cell proliferation in SIV-infected natural hosts is the main factor behind protection from disease progression [2,15]. It is also known that depletion of CD20 cells in AGMs does not alter the course of virus replication or cause the AGMs to progress to AIDS, thus indicating that humoral immune responses are not vital in controlling virus replication in natural hosts [16].

There are several lines of evidence, accumulated from the study of pathogenic infections that cell-mediated immune responses may control HIV and SIV replication. Thus, numerous studies have reported that HIV and SIV "elite controllers" that effectively control viral replication and disease progression [17-22] have preserved functional CD8^+ ^T cell response against lentiviral proteins [17-19]. CD8^+ ^cell depletion studies in RMs during the acute and chronic SIV infection have led to significant increases in SIV VLs and rapid disease progression [23-26]. Moreover, vaccine studies that have demonstrated protection in RMs challenged with pathogenic SIV strains have utilized CD8^+ ^depletion to identify the factors responsible for protection against the challenge virus. In these studies, the depletion of CD8^+ ^T cells resulted in increased viral replication of either the challenge strain or the vaccine strain [27-33]. Finally, the importance of CD8^+ ^cells in lowering VLs during antiretroviral therapy has also been reported in SIV-infected RMs [34,35].

In spite of the overwhelming evidence of the active role that CD8^+ ^cells may play during SIV infection in RMs, the role of immune responses in controlling SIV infection in natural hosts is still under debate. It has been shown in AGMs that there is a variable CD8^+ ^T cell response against SIV antigens such as Gag and Env [36,37]. During acute SIVsmm infection, SMs showed lower CD8^+ ^T cell proliferation compared to RMs, which was interpreted as evidence that SMs do not have robust T cell responses [38]. Moreover, in chronically infected SMs, the same group reported that the control of viral replication is independent of cellular immune responses [39]. However, another group reported that a correlation can be established between the SIVsmm-specific CD8^+ ^T cell responses and VLs, arguing that CD8^+ ^T cells do play a role in natural host species [40] and that cytotoxic T lymphocyte escape mutations occur during SIVsmm infection in SMs, pointing to a role of cell-mediated immunity in controlling viral replication [41,42]. Immune cell depletion studies also reported contradictory results. In one study it was reported that *in vivo *CD8^+ ^cell depletion had no impact on SIV replication in SMs. In that study, increases in viral replication were observed after CD8^+ ^cell depletion, but the authors interpreted them as being related to increases in activated and proliferating CD4^+ ^T cells rather than to the ablation of cell-mediated immunity [43]. Two recent studies that combined CD8 and CD20 cell depletion in two different species of AGMs reported a trend toward a prolongation in peak viremia that was controlled with the rebound of the CD8^+ ^T cells, and had no impact on the course of SIVagm infection [44,45].

In this study, we performed experimental *in vivo *CD8^+ ^cell depletion in AGMs during acute SIV infection. We report that CD8^+ ^cell depletion results in a lack of control of acute viral replication, thus pointing out a role for cellular immune responses in the partial control of acute viral replication in natural hosts. However, this lack of control of viral replication did not result in rapid disease progression, most probably because of the short duration of CD8^+ ^cell depletion. Thus, our results support the paradigm in which cell-mediated immune responses are involved in a partial control of viral replication in natural hosts below levels that may trigger the factors responsible for disease progression (i.e. excessive immune activation, cell proliferation and apoptosis).

## Results

### Clinical and serological data

Nine AGMs were infected with SIVagm.sab and seroconverted by day 21-28 p.i. (data not shown). Four were depleted of CD8 cells by use of an anti-CD8 mAb, while 5 served as controls. The dynamics of serological markers of SIVagm.sab infection was similar between CD8-depleted AGMs and controls (data not shown). None of the animals developed fever after infection with SIVagm.sab. No clinical signs of primary infection, weight loss, opportunistic infection or increase in size of LNs were observed during the acute phase of infection or later on. Animals were monitored up to day 225 p.i. when they were euthanized. No clinical or pathological sign of AIDS was observed at the necropsy.

### cM-T807 mAb treatment successfully depleted CD8^+ ^cells in blood and LNs, but lead only to incomplete depletion and down regulation of CD8 on cells in the intestine

**Figure 1:**
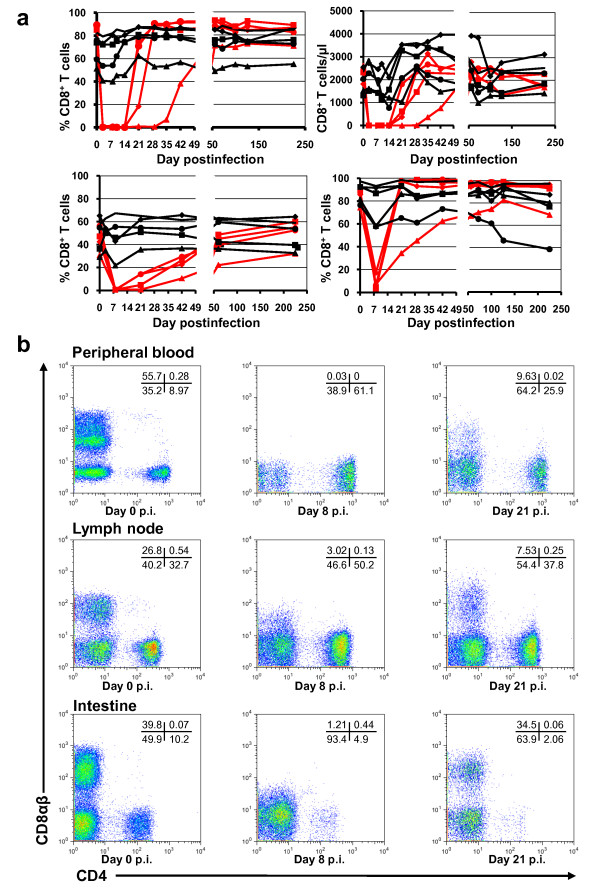
**Effect of cM-T807 administration on CD8^+ ^T cells of African green monkeys**. a. As shown by flow cytometry analysis, the CD8-depleting antibody induced complete depletion in peripheral blood for 14-21 days. Both percentage (upper left panel) and absolute (upper right panel) CD8^+ ^T cell counts are shown. In the LN, the depletion, although complete was shorter than in periphery (lower left panel), while in the intestine, only a transient, incomplete depletion was observed (lower right panel). Red symbols and lines denote CD8-depleted monkeys. Black symbols and lines denote the control monkeys. b. Flow-cytometry plots of CD4^+ ^and CD8^+ ^populations (gated on CD3^+^) in peripheral blood, lymph nodes and intestine demonstrate that administration of CD8-depleting mAb determined depletion in periphery and lymphoid tissue and likely induced down regulation/blocking of CD8 molecule rather than CD8^+ ^cell depletion in the intestine. Thus, in monkeys treated with cM-T807 mAb (lower panels), downregulation of CD8 cells in the gut is suggested by the massive increase in the double negative population at the time of maximum depletion (day 8 p.i.) compared to baseline (day 0 p.i.). With the rebound of CD8^+ ^cells, this double negative population vanishes, as illustrated here by a plot on samples collected at day 28 p.i.

Administration of 50 mg/kg of cM-T807 at day 0, followed by a 10 mg/kg dose at days 6 and 13 p.i resulted in complete depletion of peripheral CD8^+ ^T cells that was maintained in the peripheral blood through day 14 p.i. in three AGMs, while depletion lasted for 28 days in the fourth AGM receiving the anti-CD8 mAb (Figure 1a, upper panels). With the exception of partial, transient CD8^+ ^T cell decline corresponding to the peak of viral replication, the levels of CD8^+ ^T cells were stable in controls (Figure 1a, upper panels).

In the LNs, depletion of CD8^+ ^cells was not as sustained as that observed in peripheral blood and lasted less than 14 days in all animals (Figure 1a, lower left panel). In the intestine, assessment of CD8^+ ^T cell depletion using an anti-CD8αα MAb showed that CD8^+ ^T cell depletion was transient and incomplete. CD8^+ ^cells could be detected in the intestine as early as day 8 p.i. (Figure 1a, lower right panel). This is in agreement with previous studies in RMs, for which mucosal CD8 depletion was incomplete even at high doses of cM-T807 [23,33]. Flow cytometry analysis of the efficacy of CD8^+ ^T cell depletion using an anti-CD8αβ MAb confirmed complete depletion in periphery and lymphoid tissue, and the downregulation of CD8^+ ^cells in the intestine. Thus, in contrast to control animals, in which there was no significant change in the CD8^+ ^T cell population during the follow-up (data not shown), in CD8-depleted monkeys, cM-T807 administration resulted in a significant change of CD8^+ ^T cells (Figure 1b, lower panels). At day 8 p.i., flow cytometry analysis failed to identify CD8^+ ^T cells in the intestine of cM-T807-treated monkeys. However, one should note that at this time point, there was a dramatic increase of the double negative cell population when compared to both day 0 p.i. and day 28 p.i. (Figure 1b, lower panels). This observation strongly suggests a down regulation of CD8 expression rather than a true complete CD8^+ ^cell depletion at the mucosal sites.

**Figure 2:**
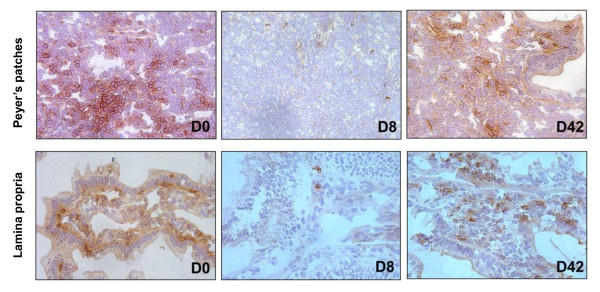
**Immunohostochemistry assessment of the efficacy of CD8 depletion on jejunum**. Samples from CD8-depleted AGMs were collected at the baseline, at day 8 p.i. and at day 42.p.i. As illustrated, CD8^+ ^cells from both Peyer patches (PP) and lamina propria (LP) are depleted/down regulated.

IHC staining for CD8^+ ^cells in intestinal tissue showed a significant decline of CD8-expression by cells in both the Peyer's patches (Figure 2, upper panels) and lamina propria (Figure 2, lower panels) of the jejunum at day 8 p.i., compared to days 0 and 42 p.i. Note that the reduction in CD8 expression observed by IHC may be the result of the blockage of CD8 by the cM-T807 antibody, in agreement with previous report indicating that cM-T807 could also block the CD8 marker which would result in a loss of CD8^+ ^cell function in this compartment [23,33] and that residual CD8^+ ^cells in the intestine may be functionally inactive.

**Figure 3:**
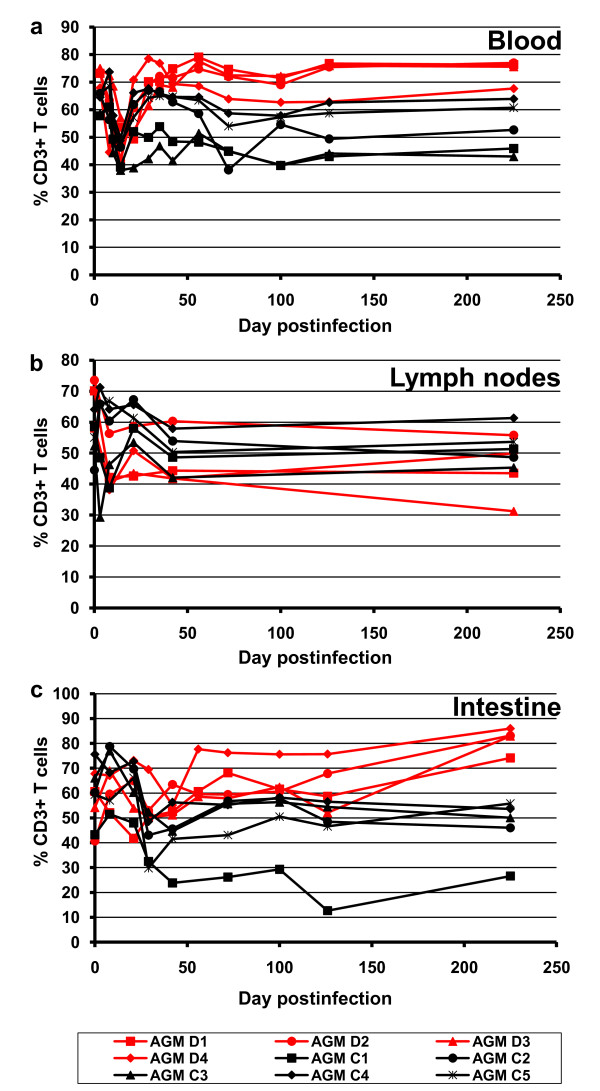
**Dynamics of CD3^+ ^cells demonstrated significant decreases in blood (a) and LNs (b), suggestive for CD8^+ ^cell depletion at these sites**. Conversely, no significant dynamics of the CD3^+ ^cells were observed in the intestine (c), thus confirming CD8 down regulation at this site. Black symbols and lines denote the control monkeys. Red symbols and lines denote CD8-depleted monkeys.

A decrease in the both the percentages (Figure 3a) and absolute numbers (data not shown) of CD3^+ ^cells was observed in both peripheral blood and LNs (Figure 3b), thus providing further evidence that CD8^+ ^cell depletion indeed occurred at least partially in lymphoid tissues. In contrast, the CD3^+ ^cell levels in the intestine were stable in the cM-T807 treated animals and were in a similar range as in control animals (Figure 3c), thus pointing towards either a downregulation of the CD8^+ ^marker or a blocking by the cM-T807 antibody at mucosal sites.

### Impact of CD8^+ ^cell depletion on the control of SIVagm.sab92018 replication

**Figure 4:**
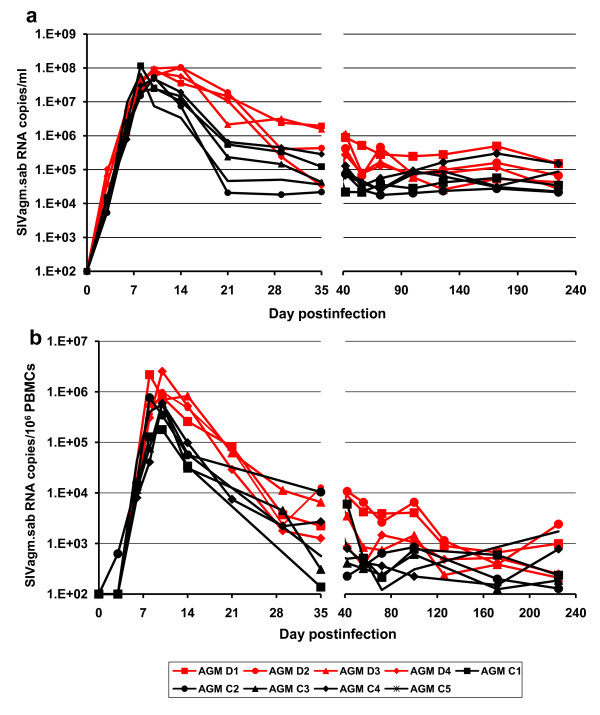
**Dynamics of SIVagm.sab plasma (a) and PBMC (b) vRNA loads in cM-T807-treated AGMs and control monkeys**. In CD8-depleted AGMs there was a delay in the control of acute viral replication. Black symbols and lines denote the control monkeys. Red symbols and lines denote CD8-depleted monkeys.

*The *VLs peaked later in CD8-depleted AGMs (days 10-14 p.i.) than in controls (days 8 and 10 p.i.). Peak VLs were similar between the two groups, ranging from 10^7 ^to 10^8 ^SIV RNA copies/ml (average: 9.37 ± 1.36 × 10^7 ^*versus *6.13 ± 3.41 × 10^7 ^SIV RNA copies/ml, p = 0.078) (Figure 4a). There was a delay in the post-peak control of viral replication in the CD8-depleted AGMs compared to controls and, at day 21 p.i., VLs ranged from 10^6 ^to 10^7 ^SIVagm.sab RNA copies/ml (average: 1.16 ± 0.71 × 10^7 ^SIVagm.sab RNA copies/ml) and were of 10^4 ^to 10^6 ^SIVagm.sab RNA copies/ml (average: 3.02 ± 2.9 × 10^5 ^SIVagm.sab RNA copies/ml) in controls (p = 0.016) (Figure 4a). Analysis of the area under the curve of the logarithm of VL showed that cM-T807-treated AGMs had higher viral replication than controls at least up to day 29 p.i. (p = 0.016). VLs of cM-T807-treated AGMs and controls were then similar at later time points (i.e., 1.14 ± 0.89 × 10^5 ^*vs *0.69 ± 0.35 × 10^5 ^SIVagm.sab RNA copies/ml at day 100 p.i.) (Figure 4a), coincidental with the appearance of CD8^+ ^cells (Figure 1a).

SIVagm replication in PBMC showed a similar pattern, with significantly higher VLs in cM-T807-treated AGMs compared to controls during early acute infection (Figure 4b), as measured by the area under the logarithm of the viral levels up to day 29 p.i. (p = 0.016). With the rebound of CD8^+ ^cells, similar control of viral replication was observed in both groups (Figure 4b).

### Effects of CD8^+ ^cell depletion on other immune cell subsets

**Figure 5:**
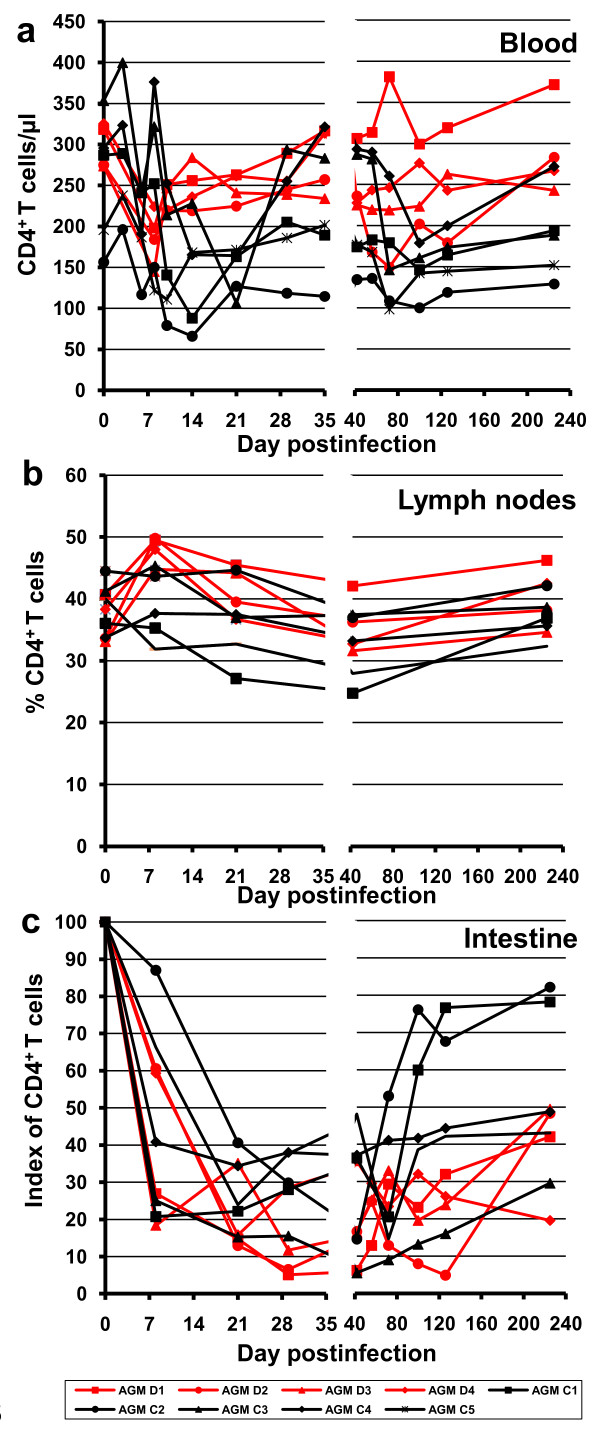
**Changes in CD4^+ ^T cells in blood (a), lymph nodes (b) and intestine (c) in CD8-depleted AGMs (red lines and dots) and control monkeys (black lines and dots)**. The index of mucosal CD4 T cells is calculated as the proportion of CD4^+ ^CD3^+ ^T cells at different time points relative to the baseline levels. This index illustrates the degree of CD4^+ ^T cell depletion.

When the absolute counts of peripheral CD4^+ ^T cells were compared between the CD8^+ ^depleted AGMs and controls, there was no significant difference between the two groups (Figure 5a). Animals in both groups experienced a transient depletion of CD4^+ ^T cells during the acute infection, with a rebound to near baseline levels as they progressed towards the chronic infection (Figure 5a).

As expected, the depletion of CD8^+ ^T cells resulted in an increase in the percentage of CD4^+ ^T cells in the cM-T807 treated animals in the peripheral blood (data not shown) and LNs (Figure 5b), as measured by flow cytometry. Moreover, in the post-acute infection, the absolute CD4^+ ^T cell counts were slightly higher in the peripheral blood of CD8-depleted AGMs as compared to controls, and this corresponded to higher levels of CD4^+ ^T cell proliferation (see below). However, these differences between the two groups did not reach significance levels. When we analyzed the CD4^+ ^cell population in the gut, a similar slope of CD4^+ ^T cell depletion was observed for the two AGM groups during the first month p.i. (p = 0.16) (Figure 1b and 5c). Although no significant difference in the magnitude of acute mucosal CD4^+ ^T cell depletion was observed, during the chronic infection, there was a trend toward less mucosal CD4^+ ^T cell restoration in cM-T807-treated group, probably as a result of higher viral replication for a longer period of time in this group of AGMs (Figure 5c).

### Dynamics of cells with activated phenotypes

**Figure 6:**
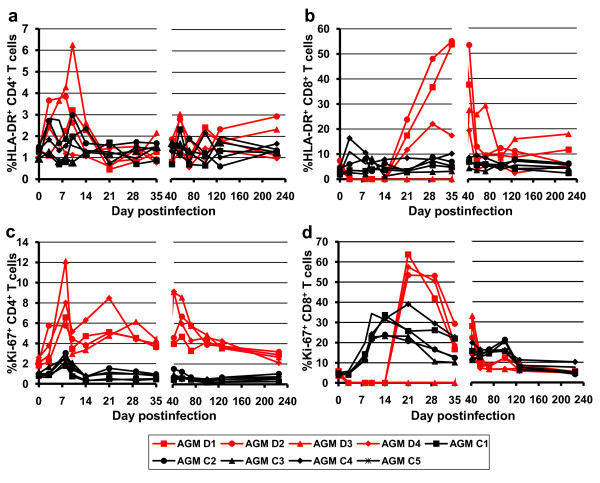
**Dynamics of CD4^+ ^and CD8^+ ^T cell immune activation (as defined by changes in the expression of MHC class II markers) (a and c) and of CD4^+ ^and CD8^+ ^T cell proliferation (as defined by changes in the expression of Ki-67) (c and d) in peripheral blood of CD8-depleted AGMs (red lines and dots) and control monkeys (black lines and dots)**. See text for further detail.

A variety of markers of immune activation, including -DR, CD25, CD69, and Ki67 was used to determine the levels of T cell immune activation during depletion of CD8^+ ^cells. The levels of activated CD4^+ ^T cells (as defined by MHC class II and CD69 expression) appeared higher in cM-T807-treated AGMs compared to controls during the acute infection in peripheral blood (Figure 6a and data not shown), although this increase did not reach significance (p > 0.19). These levels returned to near baseline levels after the set point in both groups.

The dynamics of Ki67^+ ^CD4^+ ^T cells were significantly different between cM-T807-treated AGMs and controls during both acute and chronic infection, with significantly higher expression of Ki-67 in the CD4^+ ^T cells of CD8-depleted animals (Figure 6c) throughout the follow-up.

High levels of immune activation in both peripheral blood (Figure 6b) and LNs (data not shown) were observed for CD8^+ ^T cells rebounding after the cM-T807 treatment, as measured by increased levels in all the activation markers studied: MHC class II (Figure 6b), CD69, and CD25 (data not shown). As expected, the rebounding CD8^+ ^T cells were highly proliferative, as illustrated by the high expression of Ki-67 (Figure 6d). There was no clear correlation between the levels of virus in the plasma and the levels of T cell activation and proliferation markers during the acute phase for any of the animals (e.g., one animal had higher levels of CD69^+ ^CD4^+^, CD69^+ ^CD8^+^, CD25^+ ^CD8^+^, and Ki67^+ ^CD8^+ ^cells in comparison to the other animals in the CD8^+ ^depleted group, but these did not translate into higher viremia in plasma).

**Figure 7:**
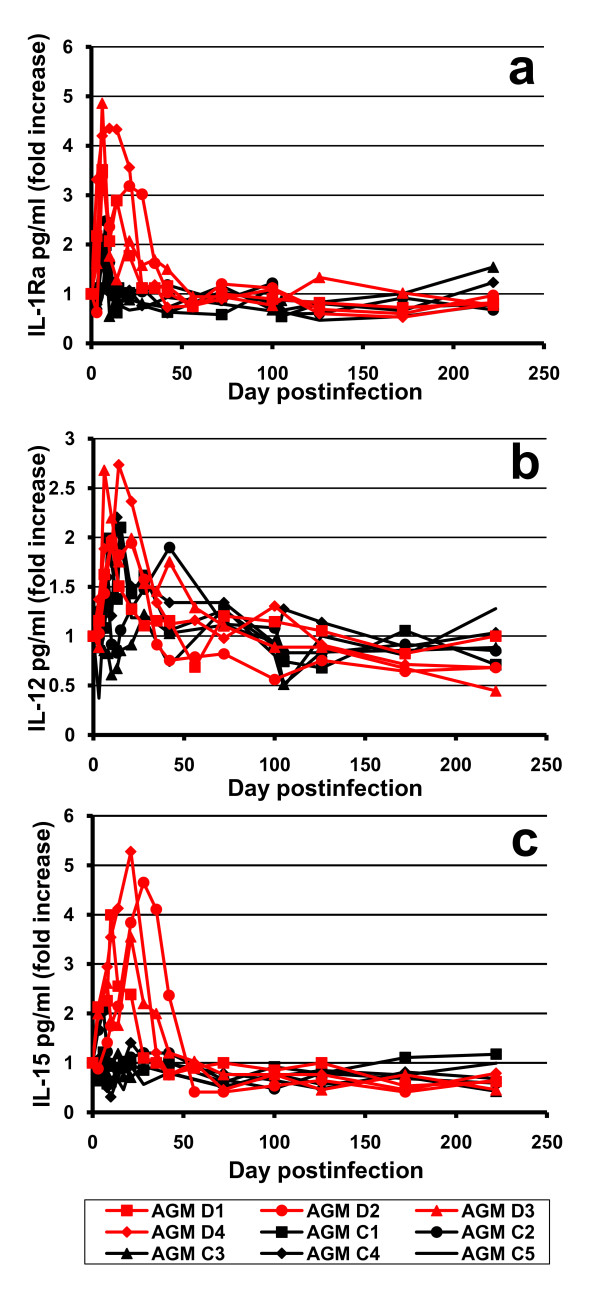
**Dynamics of plasma proinflammatory cytokine secretion: 1L-1ra (a), IL-12 (b) and IL-15 (c) in CD8-depleted AGMs (red lines and dots) and control monkeys (black lines and dots)**. See text for further detail.

These flow-cytometry data that indicated high levels of T cell activation and proliferation in cM-T807-treated AGMs were confirmed by the dynamics of proinflammatory cytokines assessed in plasma (Figure 7a-c). As illustrated, the levels of proinflammatory IL-1RA (Figure 7a) and IL-15 (Figure 7c) were significantly higher in CD8-depleted AGMs than in controls. IL-12 increased similarly in both depleted and undepleted monkeys during acute SIVagm infection (Figure 7b). These differences in cytokine levels were significant during the CD8^+ ^cell depletion (p < 0.05) and tended to persist longer than the CD8^+ ^cell depletion. Note that the higher levels of immune activation and T cell proliferation detected by immunophenotypic markers in the CD8^+ ^cell depleted group, lasted longer than those identified by the dynamics of proinflammatory cytokines.

Collectively, these results indicate that CD8^+ ^cell depletion induces significant increases in activation and proliferation of CD4^+ ^T cells. As mentioned above, the fact that the increased levels of cell activation and proliferation persist longer than the CD8^+ ^cell depletion probably indicates that the lack of control of viral replication during postacute SIVagm.sab infection of cM-T807-treated AGMs is probably mainly dependent on the availability of CD8^+ ^cells.

## Discussion

We report here that CD8^+ ^T cells are involved in the control of viral replication during acute SIV infection in natural African NHP hosts. *In vivo *CD8^+ ^cell depletion in AGMs followed by infection with SIVagm.sab resulted in a change in the pattern of acute viral replication, with the peak VL usually observed during SIV infection in RMs and natural hosts being replaced with a plateau of high VLs that lasted as long as CD8^+ ^T cells were depleted (up to 21 days p.i.). This high viral replication was controlled after the rebound of CD8^+ ^cells. Therefore, similar to previous *in vivo *CD8^+ ^depletion studies in RMs that identified the importance of CD8^+ ^T cells in controlling viral replication and disease progression [20,23-27,29,30,32,33,46,47], our results indicate that CD8^+ ^T cells are important for the control of virus replication in natural hosts, especially during the post acute infection. Our results point to similar mechanisms of controlling viral replication between pathogenic infections of humans and RMs and African NHPs that are natural hosts of SIVs. However, different from SIVmac-infected macaques in which CD8^+ ^cell depletion may result in some cases in a lack of control of viral replication and rapid disease progression [23], in SIVagm-infected AGMs VLs were controlled in all CD8-depleted monkeys at the time of the rebound of CD8^+ ^cells; and no case of disease progression was observed. One may argue that the study group was small and that in a larger study one may expect to observe a more complex clinical outcome. However, based on our previous observations, it is unlikely that natural hosts would progress to AIDS after such a short period of time of uncontrolled viral replication. We previously reported that indeed, progression to AIDS in African natural hosts is related to higher set-point replication levels [4,48], but also that African species show a remarkable resilience to high viral replication, progression to AIDS being only described after very long incubation periods [48]. Therefore, even if significant increases in VLs were observed in our study, the very short duration of CD8^+ ^cell depletion precluded disease progression in AGMs.

The administration of cM-T807 mAb successfully depleted CD8^+ ^T cells in peripheral blood and LNs and only partially and for a shorter period in the intestine. At this site, down-regulation of this cell population was also observed. Our results are similar to what was previously reported for RMs [23,33]. It is currently unknown why the anti-CD8^+ ^cM-T807 mAb is less effective in mucosal tissues, such as the intestine. Previous attempts to improve the efficacy of mucosal CD8^+ ^cell depletion through repeated, increased doses of cM-T807 failed [23], probably due to increased antibody clearance in the intestine or to antibody blockage from reaching the mucosal sites [23]. However, these studies demonstrated that the administration of cM-T807 mAb inhibited the generation of SIV-specific T cell responses [46].

Our results obtained during acute SIVagm infection of AGMs are somewhat different from the previous report on CD8^+ ^cell depletion in SMs during chronic SIVsmm infection, in which only a relatively modest increase in plasma VL (1 log or less) was observed [43] and was attributed to the increase in activating, proliferating CD4^+ ^T cells [43], rather than to the depletion of CD8^+ ^cells. However, although the increase in viral replication may have resulted from increased immune activation and proliferation of CD4^+ ^T cells due to CD8^+ ^cell depletion, one cannot definitively discard the contribution of CD8^+ ^cell depletion in the rebound of viral replication in chronically SIVsmm-infected SMs. Note that the results reported in chronically SIVsmm-infected SMs are not necessarily surprising, as CD8^+ ^depletion performed during chronic SIV infection in RMs was less effective than in SIV-uninfected animals and indicated that CD8^+ ^cell depletion resulted in modest VL increases, with no significant impact on disease progression rate [24,25].

Similar to previous reports [43], we also observed significant increased levels of Ki-67^+ ^CD4^+ ^T cells for an extended duration in the CD8^+ ^cell-depleted AGMs compared to controls. However, we believe that the increases in viral replication occurred as a result of CD8^+ ^T cell depletion rather than from increased immune activation. While agreeing that immune activation does play a significant role in controlling VLs, since we have demonstrated *in vivo *that experimental increases in immune activation correlate with increases in VLs [14], we also do not wish to downplay the significance of CD8^+ ^T cells during the post acute infection. It was impossible in this experimental setting to separate the roles of immune activation and CD8^+ ^cell depletion; however, there is evidence that can be gleaned from our study in relation to the importance of CD8 cellular immune responses in controlling acute SIVagm.sab replication: (i) VLs decreased with the reappearance of CD8^+ ^T cells, as previously reported [23,24,49], while increased levels of immune activation persisted after the control of viral replication; (ii) no clear correlation was observed between the levels of immune activation and the levels of virus replication, and (iii) finally, in a recent set of experiments performed in SIV-infected RMs, consisting of dissociation between CD8^+ ^cell depletion and immune activation of CD4^+ ^T cells (prevented through administration of an anti-IL-15 MAb), a clearer correlation could be established between CD8^+ ^cell depletion and the lack of control of viral replication [50].

It was also suggested that the increases in VLs during the CD8^+ ^cell depletion could also result from the reactivation of latent CMV, which has been suggested to occur as a result of depletion of CD8^+ ^T cells for an extended period of time [43]. However, CD8^+ ^cell depletion alone probably only results in a low-level reactivation of CMV and is not likely to contribute much to the increase in VLs observed, as research has indicated that for a high level of reactivation of CMV to occur, both the humoral and cellular immune responses need to be impaired [51].

Furthermore, because the cM-T807 antibody does not discriminate between NK cells or CD8^+ ^T cells, it could also be hypothesized that the elimination of NK cells also contributed to the rise in VLs. Because cM-T807 targets the α chain of CD8^+ ^T cells, NK cells (which contain the CD8α chain) are also depleted [20,32]. Further support of this argument comes from studies showing that during the first two weeks of infection, NK cell activity is increased in SIVmac251 RMs and decreases once the peak of viremia occurs [52], indicating that the increases in VL could also be caused by the elimination of NK cells. However, NK cells have been recently experimentally depleted in RMs during both the acute and chronic phases of SIV infection, and no impact on viral replication was observed [53,54]. Note, however, that in both these studies the NK-depleting MAb was an anti-CD16 and that not all NKs express CD16 in nonhuman primates [55]. Therefore, using CD16 as a marker to deplete NK cells may have underestimated the role of NK cells in HIV infections.

Even though our results show that depletion of CD8^+ ^cells results in higher levels of viral replication during the acute SIVagm infection of AGMs, suggesting a role of cellular immune responses in controlling infection in natural hosts, it is unlikely that cellular immunity is the sole determinant of the lack of disease progression in natural hosts. Previous studies have shown that cellular immune responses in natural hosts are not substantially different from those observed in pathogenic SIV infection in RMs [36,37,39,40]. Moreover, combined B and CD8^+ ^cell depletions (aimed at suppressing adaptive immune responses during acute SIV infection in AGMs) although delaying the partial containment of viremia, did not induce disease in AGMs [44,45], similar to the results reported here. Numerous studies have shown that during the long term co-evolution with their species-specific SIVs, natural hosts have developed a plethora of mechanisms to prevent the deleterious consequences of SIV infection; most notably the natural hosts can prevent excessive immune activation, cell proliferation and apoptosis during the chronic SIV infection. It is currently considered that the ability to fine-tune the inflammatory responses is the main mechanism through which disease progression can be prevented in natural hosts.

## Conclusions

By demonstrating a role of adaptive immune response in controlling the viral replication to levels that can be tolerated by natural hosts without disease progression, our results point to two major conclusions: first, that an effective approach for the control of HIV disease progression is not necessarily based on induction of an excess of adaptive immunity, but should be based on a balanced immune response in conjunction with the control of immune activation, cell proliferation and apoptosis. Second, the lack of disease progression in natural hosts is not due to "tolerance" of the virus by the host, but actively achieved with a large arsenal of mechanisms that act to maintain the virus at levels that can be tolerated without deleterious consequences. In light of recent vaccine failures, our results show that a successful vaccine approach for HIV most likely should consider all these mechanisms.

## Methods

### Animals

This study included nine Carribean AGMs (*Chlorocebus sabaeus*) that were housed at the Tulane National Primate Research Center (TNPRC), which is an Association for Assessment and Accreditation of Laboratory Animal Care (AAALAC) International facility. All animals were adults ranging from 6-13 years. The animals were fed and housed according to regulations set forth by the *Guide for the Care and Use of Laboratory Animals *[56] and the Animal Welfare Act. In this study, the animal experiments were approved by the Tulane University Institutional Animal Care and Use Committee (IACUC).

### Anti-CD8 Ab treatments and virus inoculation

All nine AGMs were inoculated intravenously with plasma corresponding to 300 tissue culture-infective dose (TCID_50_) of SIVagm.sab92018 [8]. Four AGMs were treated intravenously with 50 mg/kg of cM-T807, a mouse anti-human monoclonal anti-CD8 antibody, at day 0 and 10 mg/kg on days 6 and 13. The five AGMs not treated with the monoclonal antibody served as controls and were inoculated with virus only.

### Sampling of blood, LNs and intestine

Blood was collected from all the animals at 3 time points preinfection (days -35, -14, -7 p.i.), then at the time of SIVagm.sab inoculation, twice per week for the first two weeks p.i., weekly for the next four weeks, every two weeks for the next two months and then every two months, up to day 225 p.i. LN biopsies were sampled on days 0, 8, 21, 42, and 225 p.i. Intestinal endoscopies (proximal jejunum) consisting of approximately 10-15, 1-2 mm^2 ^pieces were obtained by endoscopic guided biopsy were performed on days -18, 0, 8, 21, 29, 42, 56, 72, 100, and 126 p.i. Intestinal resections (five to ten cm) were removed surgically from the animals at days -35, 8, and 42 p.i. Additional intestine pieces were obtained at necropsy. The control animals followed a similar sampling schedule.

### Isolation of lymphocytes from blood, LNs, and intestine

Within one hour of blood removal, whole blood was used for flow cytometry. Plasma was removed from the blood within two hours of sample removal, aliquoted and stored in the -80°C until VL testing was performed. PBMCs were extracted from whole blood using LSM (Organon-Technica, Durham, NC) by centrifugation. PBMCs were frozen at -80°C using freezing media containing RPMI, heat-inactivated newborn calf serum, and 10% DMSO.

Lymphocytes were separated from LNs by pressing tissue through a nylon mesh screen. Cells were filtered through nylon bags, and washed with RPMI media (Cellgro, Manassas, VA) containing 5% heat-inactivated FBS, 0.01% Penicillin-Streptomycin, 0.01% L-glutamine, and 0.01% Hepes buffer, as previously described [5,8].

Lymphocytes were separated from pinch biopsies and resections as previously described [5,14,57,58]. Mononuclear cells were separated from the blood through Ficoll density gradient centrifugation. Briefly, lymphocytes were isolated from intestinal biopsies using EDTA followed by collagenase digestion and Percoll density gradient centrifugation [5,14,58].

Within 30 minutes after separation, cells were stained for flow cytometry. Those cells not used for staining were frozen at -80°C in freezing media.

### Flow cytometry analysis of lymphocyte populations

Immunophenotyping of lymphocytes isolated from the blood, LNs and intestine was performed by using fluorescently conjugated monoclonal antibodies in a four-color staining technique. The samples were run using a FacsCalibur flow cytometer (Becton Dickinson) and the data were analyzed using Cell Quest (Becton Dickinson) and FlowJo (Tree Star, Inc). The mAbs were conjugated to FITC, PE, PerCP, or APC. The following mAbs were used for surface stains: CD3-FITC (clone no. SP34), CD8-PE (clone no. SK1), CD20-PE (clone no. L27), CD3-PerCP (clone no. SP34-2), HLA-DR-PerCP (clone no. L243), CD8α (clone no. SK1), CD4-APC (clone no. L200) (BD Bioscience) and CD8αβ (clone no. 2ST8.5H7) (Beckman Coulter). Ki-67-FITC (clone no. B56) was used for intracellular staining (BD Bioscience). All these mAbs were cross-reactive for AGMs. Whole blood was lysed using FACS lysing solution (BD Biosciences) and stained using a procedure formerly described [8]. Mononuclear cells from blood, LNs, and intestines were stained using an excess of monoclonal antibodies by incubation at 4°C for 30 minutes. Cells were then washed (400 g/7 min) with PBS and fixed with 2% paraformaldehyde. For intracellular stains, lymphocytes were fixed with 4% paraformaldehyde for 1 hour. Cells were then washed with PBS (400 g/7 min), washed with a 0.1% saponin solution (400 g/7 min), incubated with Ki-67-FITC, washed with a 0.1% saponin solution, and fixed with a 2% paraformaldehyde solution. The absolute number of peripheral lymphocytes was determined by performing cell blood counts on each blood sample.

### Analysis of anti-SIVagm.sab IgG responses

In-house SIVagm.sab-specific PIV-EIA was used for the titration of anti-gp41 and anti-V3 antibody titers, as described [59], on serial plasma or serum samples to investigate the dynamics of anti-SIVagm.sab seroconversion.

### Viral load quantification

Plasma VLs were quantified by real-time PCR, as previously described [8,9,58]. SIVagm.sab RNA loads were also quantified in mononuclear cells isolated from blood, LNs and intestinal biopsies using the same real-time PCR assay [8,9,58]. For tissue quantification, viral RNA was extracted from 5 × 10^5^-10^6 ^cells from PBMCs, LNs and intestine with RNeasy (Qiagen), and VLs were quantified as described elsewhere [8,9,58]. Simultaneous quantification of RNAse P (*RNase *P detection kit, Applied Biosystems, CA), a single copy gene with 2 copies per diploid cell, was done to normalize sample variability and allow accurate quantification of cell equivalents [16,60]. Assay sensitivity was 10 RNA copies/10^5 ^cells and 100 RNA copies per 1 ml of plasma.

### IHC

*IHC w*as performed on LNs and intestinal samples. Fresh frozen tissues in optimal cutting temperature compound (OCT) were used. Staining was done using an anti-CD8 mAb (clone SK1 BD Biosciences) and an avidin-biotin complex HRP technique (Vectastain Elite ABC kit, Vector laboratories, Burlingame, CA). Sections were visualized with DAB (Dako, Carpinteria, CA) and counterstained with hematoxylin.

### Cytokine determination

Cytokine testing in plasma was done using a sandwich immunoassay-based protein array system, the Human Cytokine 25-Plex (Biosource International, Camarillo, CA, USA), as instructed by the manufacturer and read by the Bio-Plex array reader (Bio-Rad Laboratories, Hercules, CA, USA) which uses Luminex fluorescent-bead-based technology (Luminex Corporation, Austin, TX, USA).

### Statistical analysis of data

Data comparisons between AGMs depleted of CD8^+ ^T cells and controls were done using two-tailed non-parametric tests (Mann-Whitney). These tests included analyses of VL, HLA-DR, CD69 and Ki67 over the first weeks p.i., when the effects of infection were most pronounced (see "Results"). These variables were analyzed estimating the areas under the curve by numerical integration of a spline interpolation of the data (the logarithm for VL to avoid over-emphasizing the peak of viral infection) using Mathematica 6.0 (Wolfram Research Inc, IL). The depletion of CD4^+ ^T-cells was analyzed by linear mixed effects models over the period indicated. Where needed, appropriate transformations were applied, so that the assumptions of homoscedasticity and normality of residuals were met. Significance was assessed at the p = 0.05 level, and analyses were performed using S-Plus 2000 (MathSoft Inc, MA).

## List of abbreviations

AGM: African green monkey; SIV: simian immunodeficiency virus; VL: viral load; LN: lymph node; NHP: nonhuman primate; RM: rhesus macaque; SM: sooty mangabey; p.i.: postinfection; IHC: immunohistochemistry; CMV: cytomegalovirus; NK: natural killer; PBMCs: peripheral blood mononuclear cells; LSM: lymphocyte separation media; mAb: monoclonal antibody; FITC: fluorescein isothiocyanate; PE: phycoerythrin; PerCP: perdinin chlorophyll protein; APC: allophycocyanin; PBS: phosphate buffer saline; PIV: EIA-primate immunodeficiency virus enzyme immunoassay; DMSO: dimethylsulfoxide; MHC: Major histocompatibility complex.

## Competing interests

The authors declare that they have no competing interests.

## Authors' contributions

TG performed the animal work, cell separation, flow cytometry staining, data analyses and drafted the manuscript. RMR participated in the design of the study and performed the statistical analysis. RG carried out the nucleic acid extraction and VL quantification, as well as cytokine testing. JD coordinated the animal work and administrated the anti-CD8 antibody. DM performed RNA/DNA extraction from tissues, VL quantification and carried the immunoassays. IP and CA conceived of the study, participated in its design and coordination and helped to draft the manuscript. All authors read and approved the final manuscript.

## References

[B1] Pandrea I Apetrei C Gordon S Barbercheck J Dufour J Bohm R Sumpter B Roques P Marx PA Hirsch VM Kaur A Lackner AA Veazey RS Silvestri G Paucity of CD4+CCR5+ T cells is a typical feature of natural SIV hosts Blood 2007 109 1069 1076 10.1182/blood-2006-05-024364 17003371 PMC1785133

[B2] Pandrea I Sodora DL Silvestri G Apetrei C Into the wild: simian immunodeficiency virus (SIV) infection in natural hosts Trends Immunol 2008 29 419 428 10.1016/j.it.2008.05.004 18676179 PMC2840226

[B3] Pandrea I Silvestri G Onanga R Veazey RS Marx PA Hirsch VM Apetrei C Simian immunodeficiency viruses replication dynamics in African non-human primate hosts: common patterns and species-specific differences J Med Primatol 2006 35 194 201 10.1111/j.1600-0684.2006.00168.x 16872282

[B4] Apetrei C Gautam R Sumpter B Carter AC Gaufin T Staprans SI Else J Barnes M Cao R Jr Garg S Milush JM Sodora DL Pandrea I Silvestri G Virus-subtype specific features of natural SIVsmm infection in sooty mangabeys J Virol 2007 81 7913 7923 10.1128/JVI.00281-07 17507488 PMC1951324

[B5] Pandrea I Gautam R Ribeiro R Brenchley JM Butler IF Pattison M Rasmussen T Marx PA Silvestri G Lackner AA Perelson AS Douek DC Veazey RS Apetrei C Acute loss of intestinal CD4+ T cells is not predictive of SIV virulence J Immunol 2007 179 3035 3046 17709518 10.4049/jimmunol.179.5.3035 PMC2367134

[B6] Onanga R Kornfeld C Pandrea I Estaquier J Souquiere S Rouquet P Mavoungou VP Bourry O M'Boup S Barre-Sinoussi F Simon F Apetrei C Roques P Müller-Trutwin MC High levels of viral replication contrast with only transient changes in CD4+ and CD8+ cell numbers during the early phase of experimental infection with simian immunodeficiency virus SIVmnd-1 in *Mandrillus sphinx* J Virol 2002 76 10256 10263 10.1128/JVI.76.20.10256-10263.2002 12239301 PMC136548

[B7] Onanga R Souquiere S Makuwa M Mouinga-Ondeme A Simon F Apetrei C Roques P Primary simian immunodeficiency virus SIVmnd-2 infection in mandrills (*Mandrillus sphinx*) J Virol 2006 80 3303 3309 10.1128/JVI.80.7.3301-3309.2006 PMC1440382 16537597

[B8] Pandrea I Apetrei C Dufour J Dillon N Barbercheck J Metzger M Jacquelin B Bohm R Marx PA Barre-Sinoussi F Hirsch VM Müller-Trutwin MC Lackner AA Veazey RS Simian immunodeficiency virus (SIV) SIVagm.sab infection of Caribbean African green monkeys: New model of the study of SIV pathogenesis in natural hosts J Virol 2006 80 4858 4867 10.1128/JVI.80.10.4858-4867.2006 16641277 PMC1472068

[B9] Pandrea I Kornfeld C Ploquin MJ-I Apetrei C Faye A Rouquet P Roques P Simon F Barré-Sinoussi F Müller-Trutwin MC Diop OM Impact of viral factors on very early in vivo replication profiles in SIVagm-infected African green monkeys J Virol 2005 79 6249 6259 10.1128/JVI.79.10.6249-6259.2005 15858009 PMC1091729

[B10] Pandrea I Onanga R Kornfeld C Rouquet P Bourry O Clifford S Telfer PT Abernethy K White LT Ngari P Müller-Trutwin M Roques P Marx PA Simon F Apetrei C High levels of SIVmnd-1 replication in chronically infected *Mandrillus sphinx* Virology 2003 317 119 127 10.1016/j.virol.2003.08.015 14675630

[B11] Pandrea I Onanga R Souquiere S Mouinga-Ondéme A Bourry O Makuwa M Rouquet P Silvestri G Simon F Roques P Apetrei C Paucity of CD4+CCR5+ T-cells may prevent breastfeeding transmission of SIV in natural non-human primate hosts J Virol 2008 82 5501 5509 10.1128/JVI.02555-07 18385229 PMC2395173

[B12] Meythaler M Martinot A Wang Z Pryputniewicz S Kasheta M Ling B Marx PA O'Neil S Kaur A Differential CD4+ T-lymphocyte apoptosis and bystander T-cell activation in rhesus macaques and sooty mangabeys during acute simian immunodeficiency virus infection J Virol 2009 83 572 583 10.1128/JVI.01715-08 18987149 PMC2612394

[B13] Chakrabarti LA Lewin SR Zhang L Gettie A Luckay A Martin LN Skulsky E Ho DD Cheng-Mayer C Marx PA Normal T-cell turnover in sooty mangabeys harboring active simian immunodeficiency virus infection J Virol 2000 74 1209 1223 10.1128/JVI.74.3.1209-1223.2000 10627531 PMC111455

[B14] Pandrea I Gaufin T Brenchley JM Gautam R Monjure C Gautam A Coleman C Lackner AA Ribeiro R Douek DC Apetrei C Experimentally-induced immune activation in natural hosts of SIV induces significant increases in viral replication and CD4+ T cell depletion J Immunol 2008 181 6687 6691 18981083 10.4049/jimmunol.181.10.6687 PMC2695139

[B15] VandeWoude S Apetrei C Going wild: Lessons from T-lymphotropic naturally occurring lentiviruses Clin Microbiol Rev 2006 19 728 762 10.1128/CMR.00009-06 17041142 PMC1592692

[B16] Gaufin T Pattison M Gautam R Stoulig C Dufour J MacFarland J Mandell D Tatum C Marx M Ribeiro RM Montefiori D Apetrei C Pandrea I Effect of B cell depletion on viral replication and clinical outcome of SIV infection in a natural host J Virol 2009 83 10347 10357 10.1128/JVI.00880-09 19656874 PMC2753117

[B17] Klein MR van Baalen CA Holwerda AM Kerkhof SR Garde Bende RJ Keet IP Eeftinck-Schattenkerk JK Osterhaus AD Schuitemaker H Miedema F Kinetics of Gag-specific cytotoxic T lymphocyte responses during the clinical course of HIV-1 infection: a longitudinal analysis of rapid progressors and long-term asymptomatics J Exp Med 1995 181 1365 1372 10.1084/jem.181.4.1365 7699324 PMC2191947

[B18] Rinaldo C Huang XL Fan ZF Ding M Beltz L Logar A Panicali D Mazzara G Liebmann J Cottrill M High levels of anti-human immunodeficiency virus type 1 (HIV-1) memory cytotoxic T-lymphocyte activity and low viral load are associated with lack of disease in HIV-1-infected long-term nonprogressors J Virol 1995 69 5838 5842 7637030 10.1128/jvi.69.9.5838-5842.1995 PMC189455

[B19] Betts MR Nason MC West SM De Rosa SC Migueles SA Abraham J Lederman MM Benito JM Goepfert PA Connors M Roederer M Koup RA HIV nonprogressors preferentially maintain highly functional HIV-specific CD8+ T cells Blood 2006 107 4781 4789 10.1182/blood-2005-12-4818 16467198 PMC1895811

[B20] Friedrich TC Valentine LE Yant LJ Rakasz EG Piaskowski SM Furlott JR Weisgrau KL Burwitz B May GE Leon EJ Soma T Napoe G Capuano SV Wilson NA Watkins DI Subdominant CD8+ T-cell responses are involved in durable control of AIDS virus replication J Virol 2007 81 3465 3476 10.1128/JVI.02392-06 17251286 PMC1866056

[B21] Loffredo JT Maxwell J Qi Y Glidden CE Borchardt GJ Soma T Bean AT Beal DR Wilson NA Rehrauer WM Lifson JD Carrington M Watkins DI Mamu-B*08-positive Macaques Control Simian Immunodeficiency Virus Replication J Virol 2007 81 8827 8832 10.1128/JVI.00895-07 17537848 PMC1951344

[B22] Yant LJ Friedrich TC Johnson RC May GE Maness NJ Enz AM Lifson JD O'Connor DH Carrington M Watkins DI The high-frequency major histocompatibility complex class I allele Mamu-B*17 is associated with control of simian immunodeficiency virus SIVmac239 replication J Virol 2006 80 5074 5077 10.1128/JVI.80.10.5074-5077.2006 16641299 PMC1472056

[B23] Veazey RS Acierno PM McEvers KJ Baumeister SH Foster GJ Rett MD Newberg MH Kuroda MJ Williams K Kim EY Wolinsky SM Rieber EP Piatak M Jr Lifson JD Montefiori DC Brown CR Hirsch VM Schmitz JE Increased loss of CCR5+ CD45RA- CD4+ T cells in CD8+ lymphocyte-depleted Simian immunodeficiency virus-infected rhesus monkeys J Virol 2008 82 5618 5630 10.1128/JVI.02748-07 18367534 PMC2395171

[B24] Schmitz JE Kuroda MJ Santra S Sasseville VG Simon MA Lifton MA Racz P Tenner-Racz K Dalesandro M Scallon BJ Ghrayeb J Forman MA Montefiori DC Rieber EP Letvin NL Reimann KA Control of viremia in simian immunodeficiency virus infection by CD8+ lymphocytes Science 1999 283 857 860 10.1126/science.283.5403.857 9933172

[B25] Jin X Bauer DE Tuttleton SE Lewin S Gettie A Blanchard J Irwin CE Safrit JT Mittler J Weinberger L Kostrikis LG Zhang L Perelson AS Ho DD Dramatic rise in plasma viremia after CD8+ T cell depletion in simian immunodeficiency virus-infected macaques J Exp Med 1999 189 991 998 10.1084/jem.189.6.991 10075982 PMC2193038

[B26] Metzner KJ Jin X Lee FV Gettie A Bauer DE Di Mascio M Perelson AS Marx PA Ho DD Kostrikis LG Connor RI Effects of in vivo CD8+ T cell depletion on virus replication in rhesus macaques immunized with a live, attenuated simian immunodeficiency virus vaccine J Exp Med 2000 191 1921 1931 10.1084/jem.191.11.1921 10839807 PMC2213531

[B27] Metzner KJ Moretto WJ Donahoe SM Jin X Gettie A Montefiori DC Marx PA Binley JM Nixon DF Connor RI Evaluation of CD8+ T-cell and antibody responses following transient increased viraemia in rhesus macaques infected with live, attenuated simian immunodeficiency virus J Gen Virol 2005 86 3375 3384 10.1099/vir.0.81206-0 16298985

[B28] Willey RL Byrum R Piatak M Kim YB Cho MW Rossio JL Jr Bess J Jr Igarashi T Endo Y Arthur LO Lifson JD Martin MA Control of viremia and prevention of simian-human immunodeficiency virus-induced disease in rhesus macaques immunized with recombinant vaccinia viruses plus inactivated simian immunodeficiency virus and human immunodeficiency virus type 1 particles J Virol 2003 77 1163 1174 10.1128/JVI.77.2.1163-1174.2003 12502833 PMC140830

[B29] Amara RR Ibegbu C Villinger F Montefiori DC Sharma S Nigam P Xu Y McClure HM Robinson HL Studies using a viral challenge and CD8 T cell depletions on the roles of cellular and humoral immunity in the control of an SHIV-89.6P challenge in DNA/MVA-vaccinated macaques Virology 2005 343 246 255 10.1016/j.virol.2005.08.027 16185742

[B30] Rasmussen RA Hofmann-Lehmann R Li PL Vlasak J Schmitz JE Reimann KA Kuroda MJ Letvin NL Montefiori DC McClure HM Ruprecht RM Neutralizing antibodies as a potential secondary protective mechanism during chronic SHIV infection in CD8+ T-cell-depleted macaques Aids 2002 16 829 838 10.1097/00002030-200204120-00002 11919484

[B31] Vaccari M Mattapallil J Song K Tsai WP Hryniewicz A Venzon D Zanetti M Reimann KA Roederer M Franchini G Reduced protection from simian immunodeficiency virus SIVmac251 infection afforded by memory CD8+ T cells induced by vaccination during CD4+ T-cell deficiency J Virol 2008 82 9629 9638 10.1128/JVI.00893-08 18667509 PMC2546957

[B32] Genesca M Skinner PJ Hong JJ Li J Lu D McChesney MB Miller CJ With minimal systemic T-cell expansion, CD8+ T Cells mediate protection of rhesus macaques immunized with attenuated simian-human immunodeficiency virus SHIV89.6 from vaginal challenge with simian immunodeficiency virus J Virol 2008 82 11181 11196 10.1128/JVI.01433-08 18787003 PMC2573271

[B33] Malkevitch NV Patterson LJ Aldrich MK Wu Y Venzon D Florese RH Kalyanaraman VS Pal R Lee EM Zhao J Cristillo A Robert-Guroff M Durable protection of rhesus macaques immunized with a replicating adenovirus-SIV multigene prime/protein boost vaccine regimen against a second SIVmac251 rectal challenge: role of SIV-specific CD8+ T cell responses Virology 2006 353 83 98 10.1016/j.virol.2006.05.012 16814356

[B34] Van Rompay KK Singh RP Pahar B Sodora DL Wingfield C Lawson JR Marthas ML Bischofberger N CD8+-cell-mediated suppression of virulent simian immunodeficiency virus during tenofovir treatment J Virol 2004 78 5324 5337 10.1128/JVI.78.10.5324-5337.2004 15113912 PMC400346

[B35] Lifson JD Rossio JL Piatak M Jr Parks T Li L Kiser R Coalter V Fisher B Flynn BM Czajak S Hirsch VM Reimann KA Schmitz JE Ghrayeb J Bischofberger N Nowak MA Desrosiers RC Wodarz D Role of CD8(+) lymphocytes in control of simian immunodeficiency virus infection and resistance to rechallenge after transient early antiretroviral treatment J Virol 2001 75 10187 10199 10.1128/JVI.75.21.10187-10199.2001 11581387 PMC114593

[B36] Zahn RC Rett MD Korioth-Schmitz B Sun Y Buzby AP Goldstein S Brown CR Byrum RA Freeman GJ Letvin NL Hirsch VM Schmitz JE Simian Immunodeficiency Virus (SIV)-specific CD8+ T cell responses in chronically SIVagm-infected vervet African green monkeys J Virol 2008 82 11577 11588 10.1128/JVI.01779-08 18829748 PMC2583661

[B37] Lozano Reina JM Favre D Kasakow Z Mayau V Nugeyre MT Ka T Faye A Miller CJ Scott-Algara D McCune JM Barré-Sinoussi F Diop OM Müller-Trutwin MC Gag p27-specific B- and T-cell responses in Simian immunodeficiency virus SIVagm-infected African green monkeys J Virol 2009 83 2770 2777 10.1128/JVI.01841-08 19109377 PMC2648264

[B38] Silvestri G Fedanov A Germon S Kozyr N Kaiser WJ Garber DA McClure H Feinberg MB Staprans SI Divergent host responses during primary simian immunodeficiency virus SIVsm infection of natural sooty mangabey and nonnatural rhesus macaque hosts J Virol 2005 79 4043 4054 10.1128/JVI.79.7.4043-4054.2005 15767406 PMC1061583

[B39] Dunham R Pagliardini P Gordon S Sumpter B Engram J Moanna A Lawson B McClure HM Xian-Xu H Ibegbu C Easley K Katz N Pandrea I Apetrei C Sodora DL Staprans SI Feinberg MB Silvestri G The AIDS-resistance of naturally SIV-infected sooty mangabeys is independent of cellular immunity to the virus Blood 2006 108 209 217 10.1182/blood-2005-12-4897 16522814 PMC1895834

[B40] Wang Z Metcalf B Ribeiro RM McClure H Kaur A Th-1-type cytotoxic CD8+ T-lymphocyte responses to simian immunodeficiency virus (SIV) are a consistent feature of natural SIV infection in sooty mangabeys J Virol 2006 80 2771 2783 10.1128/JVI.80.6.2771-2783.2006 16501086 PMC1395440

[B41] Kaur A Alexander L Staprans SI Denekamp L Hale CL McClure HM Feinberg MB Desrosiers RC Johnson RP Emergence of cytotoxic T lymphocyte escape mutations in nonpathogenic simian immunodeficiency virus infection Eur J Immunol 2001 31 3207 3217 10.1002/1521-4141(200111)31:11<3207::AID-IMMU3207>3.0.CO;2-H 11745337

[B42] Kaur A Yang J Hempel D Gritz L Mazzara GP McClure H Johnson RP Identification of multiple simian immunodeficiency virus (SIV)-specific CTL epitopes in sooty mangabeys with natural and experimentally acquired SIV infection J Immunol 2000 164 934 943 10623842 10.4049/jimmunol.164.2.934

[B43] Barry AP Silvestri G Safrit JT Sumpter B Kozyr N McClure HM Staprans SI Feinberg MB Depletion of CD8+ cells in sooty mangabey monkeys naturally infected with simian immunodeficiency virus reveals limited role for immune control of virus replication in a natural host species J Immunol 2007 178 8002 8012 17548637 10.4049/jimmunol.178.12.8002

[B44] Schmitz JE Zahn RC Brown CR Rett MD Li M Tang H Pryputniewicz S Byrum RA Kaur A Montefiori DC Allan JS Goldstein S Hirsch VM Inhibition of adaptive immune responses leads to a fatal clinical outcome in SIV-infected pigtailed macaques but not vervet African green monkeys PLoS Pathog 2009 5 e1000691 10.1371/journal.ppat.1000691 20011508 PMC2785481

[B45] Zahn RC Rett MD Li M Tang H Korioth-Schmitz B Balachandran H White R Pryputniewicz S Letvin NL Kaur A Montefiori DC Carville A Hirsch VM Allan JS Schmitz JE Suppression of adaptive immune responses during primary SIV infection of sabaeus African green monkeys delays partial containment of viremia but does not induce disease Blood 2010 115 3070 8 10.1182/blood-2009-10-245225 20147699 PMC2858477

[B46] Schmitz JE Simon MA Kuroda MJ Lifton MA Ollert MW Vogel CW Racz P Tenner-Racz K Scallon BJ Dalesandro M Ghrayeb J Rieber EP Sasseville VG Reimann KA A nonhuman primate model for the selective elimination of CD8+ lymphocytes using a mouse-human chimeric monoclonal antibody Am J Pathol 1999 154 1923 1932 10362819 10.1016/S0002-9440(10)65450-8 PMC1866630

[B47] Matano T Shibata R Siemon C Connors M Lane HC Martin MA Administration of an anti-CD8 monoclonal antibody interferes with the clearance of chimeric simian/human immunodeficiency virus during primary infections of rhesus macaques J Virol 1998 72 164 169 9420212 10.1128/jvi.72.1.164-169.1998 PMC109361

[B48] Pandrea I Onanga R Rouquet P Bourry O Ngari P Wickings EJ Roques P Apetrei C Chronic SIV infection ultimately causes immunodeficiency in African non-human primates AIDS 2001 15 2461 2462 10.1097/00002030-200112070-00019 11826852

[B49] Kuroda MJ Schmitz JE Charini WA Nickerson CE Lifton MA Lord CI Forman MA Letvin NL Emergence of CTL coincides with clearance of virus during primary simian immunodeficiency virus infection in rhesus monkeys J Immunol 1999 162 5127 5133 10227983

[B50] Okoye A Park H Rohankhedkar M Coyne-Johnson L Lum R Walker JM Planer SL Legasse AW Sylwester AW Piatak M Lifson JD Sodora DL Villinger F Axthelm MK Schmitz JE Picker LJ Profound CD4+/CCR5+ T cell expansion is induced by CD8+ lymphocyte depletion but does not account for accelerated SIV pathogenesis J Exp Med 2009 206 1575 1588 10.1084/jem.20090356 19546246 PMC2715089

[B51] Kaur A Kassis N Hale CL Simon M Elliott M Gomez-Yafal A Lifson JD Desrosiers RC Wang F Barry P Mach M Johnson RP Direct relationship between suppression of virus-specific immunity and emergence of cytomegalovirus disease in simian AIDS J Virol 2003 77 5749 5758 10.1128/JVI.77.10.5749-5758.2003 12719568 PMC154043

[B52] Giavedoni LD Velasquillo MC Parodi LM Hubbard GB Hodara VL Cytokine expression, natural killer cell activation, and phenotypic changes in lymphoid cells from rhesus macaques during acute infection with pathogenic simian immunodeficiency virus J Virol 2000 74 1648 1657 10.1128/JVI.74.4.1648-1657.2000 10644334 PMC111639

[B53] Choi EI Reimann KA Letvin NL In vivo natural killer cell depletion during primary SIV infection in rhesus monkeys J Virol 2008 82 6758 6761 10.1128/JVI.02277-07 18434394 PMC2447079

[B54] Choi EI Wang R Peterson L Letvin NL Reimann KA Use of an anti-CD16 antibody for in vivo depletion of natural killer cells in rhesus macaques Immunology 2008 124 215 222 10.1111/j.1365-2567.2007.02757.x 18201184 PMC2566626

[B55] Oliva A Kinter AL Vaccarezza M Rubbert A Catanzaro A Moir S Monaco J Ehler L Mizell S Jackson R Li Y Romano JW Fauci AS Natural killer cells from human immunodeficiency virus (HIV)-infected individuals are an important source of CC-chemokines and suppress HIV-1 entry and replication in vitro J Clin Invest 1998 102 223 231 10.1172/JCI2323 9649576 PMC509084

[B56] National Research Council Guide for the care and use of laboratory animals 1996 National Academy Press, Washington, DC

[B57] Gaufin T Gautam R Kasheta M Ribeiro RM Ribka E Barnes M Pattison M Tatum C MacFarland J Montefiori D Kaur A Pandrea I Apetrei C Limited ability of humoral immune responses in control of viremia during infection with SIVsmmD215 strain Blood 2009 113 4250 4261 10.1182/blood-2008-09-177741 19168789 PMC2676085

[B58] Pandrea I Ribeiro RM Gautam R Gaufin T Pattison M Barnes M Monjure C Stoulig C Silvestri G Miller M Perelson AS Apetrei C Simian immunodeficiency virus SIVagm dynamics in African green monkeys J Virol 2008 82 3713 3724 10.1128/JVI.02402-07 18216122 PMC2268485

[B59] Simon F Souquiere S Damond F Kfutwah A Makuwa M Leroy E Rouquet P Berthier JL Rigoulet J Lecu A Telfer PT Pandrea I Plantier JC Barré-Sinoussi F Roques P Müller-Trutwin MC Apetrei C Synthetic peptide strategy for the detection of and discrimination among highly divergent primate lentiviruses AIDS Res Hum Retroviruses 2001 17 937 952 10.1089/088922201750290050 11461679

[B60] Gautam R Gaufin T Butler I Gautam A Barnes M Mandell D Pattison M Tatum C Macfarland J Monjure C Marx PA Pandrea I Apetrei C SIVrcm, a unique CCR2-tropic virus, selectively depletes memory CD4+ T cells in pigtailed macaques through rapid coreceptor expansion in vivo J Virol 2009 83 7894 7908 10.1128/JVI.00444-09 19493994 PMC2715763

